# A second monoclinic polymorph of 1-benzyl-*N*-methyl-1*H*-pyrrole-2-carboxamide

**DOI:** 10.1107/S1600536811030364

**Published:** 2011-08-02

**Authors:** Chang Jun Wang, Xiang Chao Zeng, Shi Hai Xu

**Affiliations:** a Department of Chemistry, Jinan University, Guangzhou, Guangdong 510632, People’s Republic of China

## Abstract

In the title compound, C_13_H_14_N_2_O, the N_pyrrole_—C(H_2_)—C—C torsion angle is −7.7 (3)° and the dihedral angle between the pyrrole and benzene rings is 83.6 (2)°. In the crystal, inter­molecular N—H⋯O hydrogen bonds link the mol­ecules into chains extending along the *c* axis. We have previously reported another polymorphic form of this title compound, which has the same space group with different cell parameters: *a* = 9.8285 (18) Å, *b* = 23.588 (4) Å, *c* = 9.9230 (17) Å, β = 90.107 (3)°, *Z* = 8 and *V* = 2300.5 (7) Å^3^ [Zeng *et al.* (2010 ▶). *Acta Cryst.* E**66**, o2051].

## Related literature

For details of the synthesis, see: Zeng *et al.* (2010 ▶); For the previously reported polymorph, see: Zeng *et al.* (2010 ▶) and for a related structure, see: Zeng *et al.* (2007 ▶).
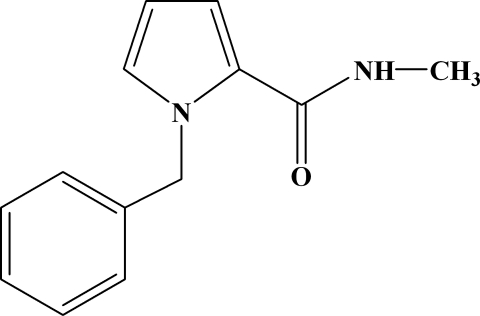


## Experimental

### 

#### Crystal data

C_13_H_14_N_2_O
*M* *_r_* = 214.26
Monoclinic, 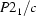

*a* = 5.4326 (4) Å
*b* = 22.5218 (16) Å
*c* = 9.7358 (5) Å
β = 101.676 (6)°
*V* = 1166.55 (13) Å^3^
*Z* = 4
Mo *K*α radiation
μ = 0.08 mm^−1^
*T* = 293 K
0.50 × 0.28 × 0.19 mm

#### Data collection

Oxford Gemini S Ultra area-detector diffractometer
Absorption correction: multi-scan (*CrysAlis PRO*; Oxford Diffraction, 2010 ▶) *T*_min_ = 0.962, *T*_max_ = 0.985
5327 measured reflections
2504 independent reflections
1597 reflections with *I* > 2σ(*I*)
*R*_int_ = 0.025

#### Refinement

*R*[*F* ^2^ > 2σ(*F* ^2^)] = 0.063
*wR*(*F* ^2^) = 0.169
*S* = 1.02
2504 reflections
150 parameters
H atoms treated by a mixture of independent and constrained refinement
Δρ_max_ = 0.17 e Å^−3^
Δρ_min_ = −0.16 e Å^−3^

### 

Data collection: *CrysAlis PRO* (Oxford Diffraction, 2010 ▶); cell refinement: *CrysAlis PRO*; data reduction: *CrysAlis PRO*; program(s) used to solve structure: *SHELXS97* (Sheldrick, 2008 ▶); program(s) used to refine structure: *SHELXL97* (Sheldrick, 2008 ▶); molecular graphics: *SHELXTL* (Sheldrick, 2008 ▶); software used to prepare material for publication: *SHELXTL*.

## Supplementary Material

Crystal structure: contains datablock(s) I, global. DOI: 10.1107/S1600536811030364/zk2014sup1.cif

Structure factors: contains datablock(s) I. DOI: 10.1107/S1600536811030364/zk2014Isup2.hkl

Supplementary material file. DOI: 10.1107/S1600536811030364/zk2014Isup3.cml

Additional supplementary materials:  crystallographic information; 3D view; checkCIF report

## Figures and Tables

**Table 1:** Hydrogen-bond geometry (Å, °)

*D*—H⋯*A*	*D*—H	H⋯*A*	*D*⋯*A*	*D*—H⋯*A*
N2—H2*A*⋯O1^i^	0.86 (3)	2.08 (3)	2.852 (2)	149 (2)

Symmetry code: (i) 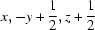.

**Fig. 1.**
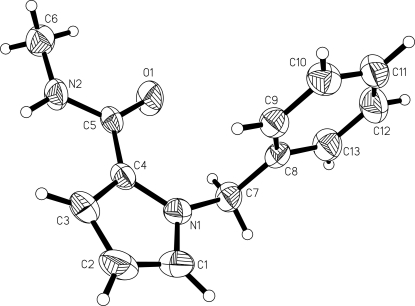
The molecular structure of the title compound, with the atom-numbering scheme. Displacement ellipsoids are drawn at the 30% probability level.

**Fig. 2.**
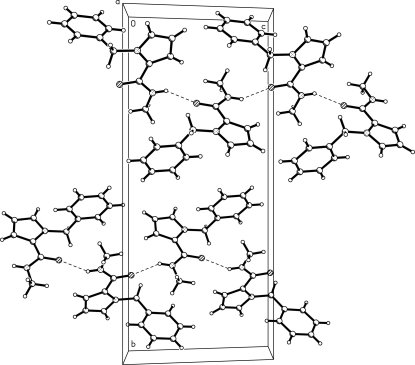
Crystal packing of (I) showing the chains formed by hydrogen bonds(dashed lines).

**Fig. 3.**
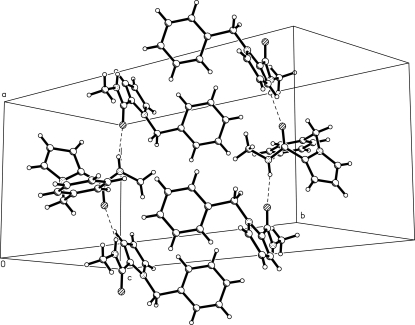
Crystal packing of the previous polymorph structure showing the chains formed by hydrogen bonds(dashed lines).

## supplementary crystallographic information

### Comment

This study is related to our previous structural investigations of 1-Benzyl-*N*-methyl-1*H*-pyrrole-2-carboxamide, I, (Zeng *et al.*, 2010) and methyl 2-(4,5-dibromo-1*H*-pyrrole-2-carboxamido)propionate (Zeng *et al.*, 2007)

In I (Fig. 1), the bond lengths and angles are almost same as those observed in the previously reported polymorphic form (Zeng *et al.*, 2010). In the crystal structure, the molecules are linked through N—H···O hydrogen bonds, forming chains extending to the *c* axis (shown in Fig. 2).

It is interesting to note here that the previously reported form was crystallized from an ethanol solution (Zeng *et al.*, 2010), while the present form was crystallized from the solution of ethanol/water (2:1 *v*/*v*). Although these two polymorphs have the same space group (*P*2_1_/*c*), their unit-cell parameters and melting point are different. For the previous one, the unit-cell parameters are *a* = 9.8285 (18), *b* = 23.588 (4), *c* = 9.9230 (17) Å, *beta* = 90.107 (3)°, with *Z* = 8, *V* = 2300.5 (7) and m.p. = 365 K. For the present one, they are 5.4326 (4), 22.5218 (16), 9.7358 (5) Å, 101.676 (6)°, *Z* = 4, *V* = 1166.55 (13) and m.p. = 360 K.

As reported, the previous polymorph structure show that the asymmetric unit of the compound contains two independent molecules, which differ in the twist of the phenyl ring: the N_pyrrole_—C(H_2_)—C—C torsion angles are -73.0 (3)° and 17.1 (3)°, respectively. And for the present one, the asymmetric unit contains just one molecule, in this molecule, the N_pyrrole_—C(H_2_)—C—C torsion angle is -7.7 (3)°, the dihedral angle between the pyrrole plane and the benzene plane is 83.60 (2)°.

The crystal packings of these two structures are differences also. In the previous polymorph structure, molecules are linked through N—H···O hydrogen bonds, generating chains extending to the *a* axis (shown in Fig. 3). And for the present one, N—H···O hydrogen bonds link molecules together to forming chains extending to the *c* axis (shown in Fig. 2).

### Experimental

The title compound was synthesized according to the literature procedure (Zeng *et al.*, 2010). The product was dissolved in the mixture of ethanol / water (2:1 *v*/*v*), colorless crystals suitable for X-ray analysis were obtained over a period of five days by slow evaporation at room temperature of the solution.

### Refinement

All non-H atoms were refined with anisotropic displacement parameters. The H atoms were positioned geometrically [C—H = 0.97Å for CH_2_, 0.96Å for CH_3_, 0.93Å for CH(aromatic) and N—H = 0.86 Å] and refined using a riding model, with *U*_iso_ = 1.2*U*_eq_ (1.5*U*_eq_ for the methyl group) of the parent atom.

### Crystal data

**Table:**

C_13_H_14_N_2_O	*D*_x_ = 1.220 Mg m^−^^3^
*M**_r_* = 214.26	Melting point: 360 K
Monoclinic, *P*2_1_/*c*	Mo *K*α radiation, λ = 0.71073 Å
*a* = 5.4326 (4) Å	Cell parameters from 1936 reflections
*b* = 22.5218 (16) Å	θ = 3.5–29.2°
*c* = 9.7358 (5) Å	µ = 0.08 mm^−^^1^
β = 101.676 (6)°	*T* = 293 K
*V* = 1166.55 (13) Å^3^	Prism, colorless
*Z* = 4	0.50 × 0.28 × 0.19 mm
*F*(000) = 456	

### Data collection

**Table:**

Oxford Gemini S Ultra area-detector diffractometer	2504 independent reflections
Radiation source: fine-focus sealed tube	1597 reflections with *I* > 2σ(*I*)
graphite	*R*_int_ = 0.025
φ and ω scans	θ_max_ = 27.0°, θ_min_ = 3.5°
Absorption correction: multi-scan (*CrysAlis PRO*; Oxford Diffraction, 2010)	*h* = −6→6
*T*_min_ = 0.962, *T*_max_ = 0.985	*k* = −28→22
5327 measured reflections	*l* = −12→11

### Refinement

**Table:**

Refinement on *F*^2^	Primary atom site location: structure-invariant direct methods
Least-squares matrix: full	Secondary atom site location: difference Fourier map
*R*[*F*^2^ > 2σ(*F*^2^)] = 0.063	Hydrogen site location: inferred from neighbouring sites
*wR*(*F*^2^) = 0.169	H atoms treated by a mixture of independent and constrained refinement
*S* = 1.02	*w* = 1/[σ^2^(*F*_o_^2^) + (0.0629*P*)^2^ + 0.3194*P*] where *P* = (*F*_o_^2^ + 2*F*_c_^2^)/3
2504 reflections	(Δ/σ)_max_ = 0.004
150 parameters	Δρ_max_ = 0.17 e Å^−^^3^
0 restraints	Δρ_min_ = −0.16 e Å^−^^3^

### Special details

**Table:**

Geometry. All e.s.d.'s (except the e.s.d. in the dihedral angle between two l.s. planes) are estimated using the full covariance matrix. The cell e.s.d.'s are taken into account individually in the estimation of e.s.d.'s in distances, angles and torsion angles; correlations between e.s.d.'s in cell parameters are only used when they are defined by crystal symmetry. An approximate (isotropic) treatment of cell e.s.d.'s is used for estimating e.s.d.'s involving l.s. planes.
Refinement. Refinement of *F*^2^ against ALL reflections. The weighted *R*-factor *wR* and goodness of fit *S* are based on *F*^2^, conventional *R*-factors *R* are based on *F*, with *F* set to zero for negative *F*^2^. The threshold expression of *F*^2^ > σ(*F*^2^) is used only for calculating *R*-factors(gt) *etc*. and is not relevant to the choice of reflections for refinement. *R*-factors based on *F*^2^ are statistically about twice as large as those based on *F*, and *R*- factors based on ALL data will be even larger.

### Fractional atomic coordinates and isotropic or equivalent isotropic displacement parameters (Å^2^)

**Table:**

	*x*	*y*	*z*	*U*_iso_*/*U*_eq_	
C5	0.0889 (4)	0.28312 (9)	0.60936 (19)	0.0517 (5)	
C4	0.2716 (4)	0.32716 (10)	0.6779 (2)	0.0543 (5)	
N1	0.4311 (3)	0.35726 (9)	0.6084 (2)	0.0652 (5)	
O1	0.0465 (4)	0.27429 (8)	0.48276 (15)	0.0877 (6)	
N2	−0.0360 (4)	0.25434 (9)	0.6916 (2)	0.0688 (6)	
C8	0.2613 (4)	0.39425 (10)	0.3667 (2)	0.0590 (6)	
C7	0.4436 (5)	0.35414 (12)	0.4599 (2)	0.0729 (7)	
H7A	0.4117	0.3135	0.4281	0.088*	
H7B	0.6127	0.3643	0.4501	0.088*	
C9	0.0783 (4)	0.42544 (11)	0.4135 (3)	0.0681 (6)	
H9	0.0665	0.4229	0.5073	0.082*	
C3	0.3192 (5)	0.34701 (11)	0.8140 (2)	0.0719 (7)	
H3	0.2398	0.3342	0.8848	0.086*	
C1	0.5712 (5)	0.39542 (13)	0.6999 (3)	0.0870 (8)	
H1	0.6916	0.4213	0.6787	0.104*	
C11	−0.0747 (6)	0.46500 (12)	0.1851 (3)	0.0847 (8)	
H11	−0.1872	0.4888	0.1244	0.102*	
C6	−0.2303 (5)	0.21166 (12)	0.6402 (3)	0.0819 (8)	
H6A	−0.3890	0.2266	0.6541	0.123*	
H6B	−0.1930	0.1750	0.6903	0.123*	
H6C	−0.2385	0.2050	0.5420	0.123*	
C12	0.1055 (6)	0.43425 (15)	0.1372 (3)	0.0962 (9)	
H12	0.1160	0.4368	0.0432	0.115*	
C10	−0.0895 (5)	0.46073 (12)	0.3229 (3)	0.0799 (7)	
H10	−0.2130	0.4816	0.3561	0.096*	
C13	0.2726 (5)	0.39933 (13)	0.2273 (3)	0.0830 (8)	
H13	0.3960	0.3787	0.1933	0.100*	
C2	0.5084 (5)	0.38987 (14)	0.8271 (3)	0.0911 (9)	
H2	0.5783	0.4107	0.9080	0.109*	
H2A	0.002 (5)	0.2599 (12)	0.781 (3)	0.092 (9)*	

### Atomic displacement parameters (Å^2^)

**Table:**

	*U*^11^	*U*^22^	*U*^33^	*U*^12^	*U*^13^	*U*^23^
C5	0.0605 (12)	0.0601 (13)	0.0343 (9)	0.0096 (10)	0.0092 (8)	0.0024 (9)
C4	0.0544 (12)	0.0661 (13)	0.0408 (10)	0.0075 (10)	0.0060 (8)	0.0031 (9)
N1	0.0503 (10)	0.0815 (14)	0.0631 (11)	0.0025 (10)	0.0095 (9)	0.0076 (10)
O1	0.1194 (15)	0.1092 (14)	0.0364 (8)	−0.0265 (12)	0.0205 (8)	−0.0120 (8)
N2	0.0875 (15)	0.0786 (14)	0.0402 (10)	−0.0144 (11)	0.0126 (10)	0.0021 (10)
C8	0.0509 (12)	0.0652 (14)	0.0648 (13)	−0.0049 (10)	0.0206 (10)	0.0075 (11)
C7	0.0605 (14)	0.0972 (19)	0.0682 (14)	0.0108 (13)	0.0297 (11)	0.0171 (13)
C9	0.0659 (14)	0.0742 (15)	0.0658 (14)	0.0021 (12)	0.0176 (11)	0.0046 (12)
C3	0.0807 (17)	0.0841 (17)	0.0457 (12)	0.0007 (14)	0.0000 (11)	−0.0033 (11)
C1	0.0592 (15)	0.097 (2)	0.096 (2)	−0.0110 (14)	−0.0053 (14)	0.0067 (17)
C11	0.091 (2)	0.0721 (17)	0.0839 (19)	0.0001 (15)	0.0011 (15)	0.0179 (14)
C6	0.0880 (19)	0.0753 (17)	0.0811 (17)	−0.0126 (14)	0.0140 (14)	0.0068 (13)
C12	0.107 (2)	0.114 (2)	0.0689 (17)	0.005 (2)	0.0211 (16)	0.0241 (17)
C10	0.0772 (17)	0.0731 (16)	0.0878 (18)	0.0086 (14)	0.0127 (14)	−0.0016 (15)
C13	0.0811 (18)	0.103 (2)	0.0721 (16)	0.0089 (16)	0.0330 (14)	0.0104 (15)
C2	0.0860 (19)	0.097 (2)	0.0756 (18)	−0.0090 (17)	−0.0194 (15)	−0.0136 (16)

### Geometric parameters (Å, °)

**Table:**

C5—O1	1.223 (2)	C3—C2	1.397 (4)
C5—N2	1.320 (3)	C3—H3	0.9300
C5—C4	1.465 (3)	C1—C2	1.355 (4)
C4—C3	1.372 (3)	C1—H1	0.9300
C4—N1	1.381 (3)	C11—C12	1.356 (4)
N1—C1	1.356 (3)	C11—C10	1.363 (4)
N1—C7	1.462 (3)	C11—H11	0.9300
N2—C6	1.440 (3)	C6—H6A	0.9600
N2—H2A	0.86 (3)	C6—H6B	0.9600
C8—C9	1.369 (3)	C6—H6C	0.9600
C8—C13	1.376 (3)	C12—C13	1.374 (4)
C8—C7	1.502 (3)	C12—H12	0.9300
C7—H7A	0.9700	C10—H10	0.9300
C7—H7B	0.9700	C13—H13	0.9300
C9—C10	1.384 (3)	C2—H2	0.9300
C9—H9	0.9300		
			
O1—C5—N2	121.1 (2)	C2—C3—H3	126.1
O1—C5—C4	122.79 (19)	C2—C1—N1	109.3 (2)
N2—C5—C4	116.09 (18)	C2—C1—H1	125.4
C3—C4—N1	107.4 (2)	N1—C1—H1	125.4
C3—C4—C5	129.7 (2)	C12—C11—C10	119.5 (3)
N1—C4—C5	122.89 (17)	C12—C11—H11	120.3
C1—N1—C4	108.3 (2)	C10—C11—H11	120.3
C1—N1—C7	123.2 (2)	N2—C6—H6A	109.5
C4—N1—C7	128.4 (2)	N2—C6—H6B	109.5
C5—N2—C6	123.2 (2)	H6A—C6—H6B	109.5
C5—N2—H2A	119.7 (19)	N2—C6—H6C	109.5
C6—N2—H2A	117.1 (18)	H6A—C6—H6C	109.5
C9—C8—C13	117.8 (2)	H6B—C6—H6C	109.5
C9—C8—C7	122.8 (2)	C11—C12—C13	120.2 (3)
C13—C8—C7	119.4 (2)	C11—C12—H12	119.9
N1—C7—C8	114.28 (19)	C13—C12—H12	119.9
N1—C7—H7A	108.7	C11—C10—C9	120.4 (3)
C8—C7—H7A	108.7	C11—C10—H10	119.8
N1—C7—H7B	108.7	C9—C10—H10	119.8
C8—C7—H7B	108.7	C12—C13—C8	121.4 (3)
H7A—C7—H7B	107.6	C12—C13—H13	119.3
C8—C9—C10	120.8 (2)	C8—C13—H13	119.3
C8—C9—H9	119.6	C1—C2—C3	107.3 (2)
C10—C9—H9	119.6	C1—C2—H2	126.3
C4—C3—C2	107.7 (2)	C3—C2—H2	126.3
C4—C3—H3	126.1		
			
O1—C5—C4—C3	171.6 (2)	C13—C8—C9—C10	0.1 (4)
N2—C5—C4—C3	−6.1 (3)	C7—C8—C9—C10	−178.4 (2)
O1—C5—C4—N1	−7.8 (3)	N1—C4—C3—C2	0.4 (3)
N2—C5—C4—N1	174.51 (19)	C5—C4—C3—C2	−179.1 (2)
C3—C4—N1—C1	−0.8 (3)	C4—N1—C1—C2	1.0 (3)
C5—C4—N1—C1	178.7 (2)	C7—N1—C1—C2	176.9 (2)
C3—C4—N1—C7	−176.4 (2)	C10—C11—C12—C13	−0.4 (5)
C5—C4—N1—C7	3.0 (3)	C12—C11—C10—C9	0.2 (4)
O1—C5—N2—C6	−0.7 (4)	C8—C9—C10—C11	−0.1 (4)
C4—C5—N2—C6	177.0 (2)	C11—C12—C13—C8	0.5 (5)
C1—N1—C7—C8	−90.4 (3)	C9—C8—C13—C12	−0.3 (4)
C4—N1—C7—C8	84.7 (3)	C7—C8—C13—C12	178.3 (3)
C9—C8—C7—N1	−7.7 (3)	N1—C1—C2—C3	−0.7 (3)
C13—C8—C7—N1	173.8 (2)	C4—C3—C2—C1	0.2 (3)

### Hydrogen-bond geometry (Å, °)

**Table:**

*D*—H···*A*	*D*—H	H···*A*	*D*···*A*	*D*—H···*A*
N2—H2A···O1^i^	0.86 (3)	2.08 (3)	2.852 (2)	149 (2)

Symmetry codes: (i) *x*, −*y*+1/2, *z*+1/2.
